# Supplementary material for: Evolution in Real-World Therapeutic Strategies for HIV Treatment: A Retrospective Study in Southern Italy, 2014–2020
Source: J Clin Med. 2021 Dec 29;11(1):161. doi: 10.3390/jcm11010161 (PMC8745745; doi:10.3390/jcm11010161)
Supplement: Supplementary file 1 [file jcm-11-00161-s001.zip › jcm-1499931-supplementary.pdf]

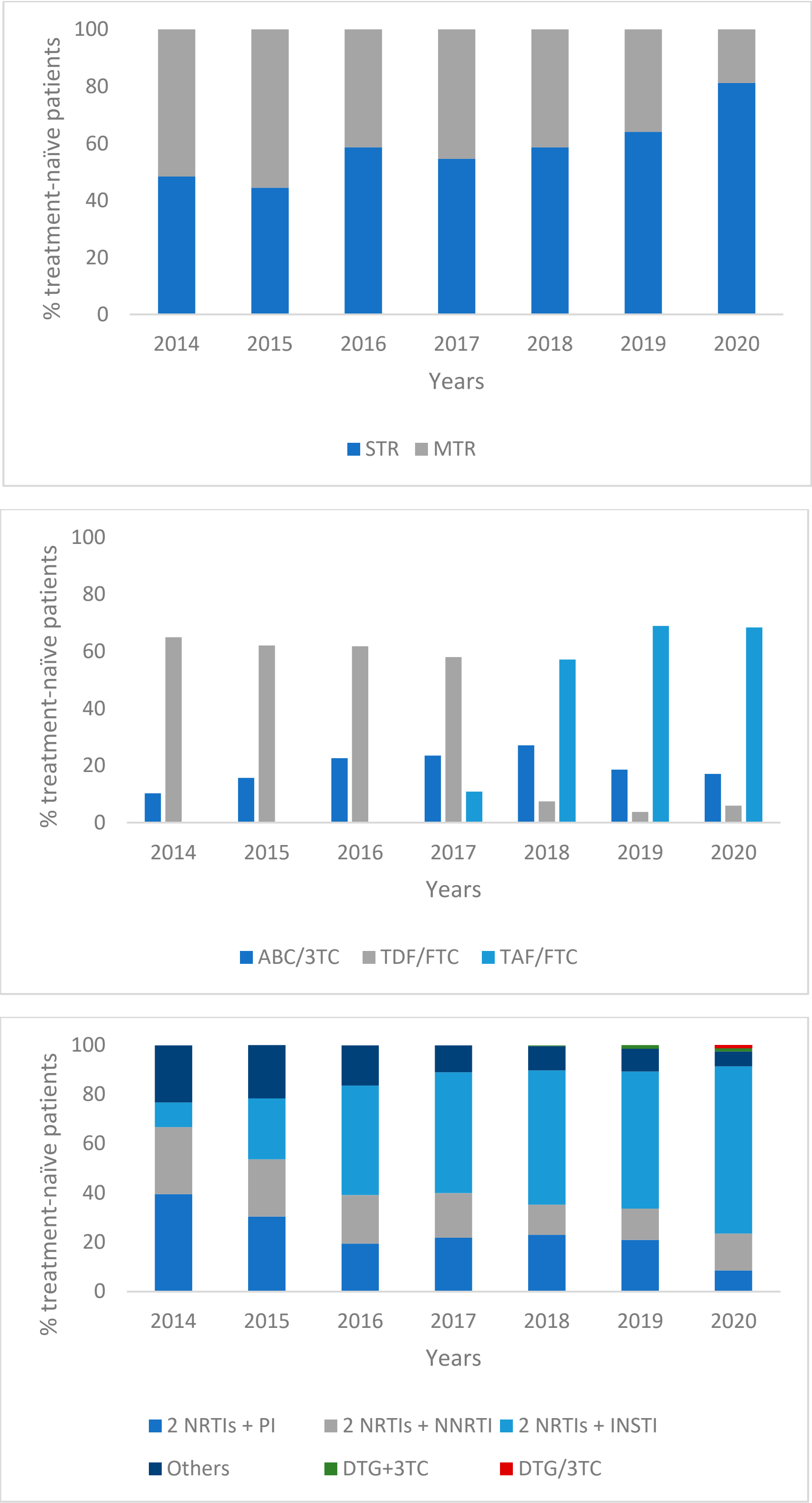

(a)

(b)

(c)

Figure S1. Distribution of treatment-naïve patients by year (2014-2020) and STR/MTR (a), NRTI backbones (b), ART regimens (c).

Abbrevation: 3TC, lamivudine; ABC, abacavir; DTG, dolutegravir; ART, combination antiretroviral treatment; FTC, emtricitabine; INSTI, integrase strand transfer inhibitor; MTR, Multiple Tablet Regimen; NRTI, nucleos(t)ide reverse transcriptase inhibitors; NNRTI, non-nucleoside reverse transcriptase inhibitors; PI, protease inhibitor; STR, Single Tablet Regimen; TAF, tenofovir alafenamide; TDF, tenofovir disoproxil fumarate.

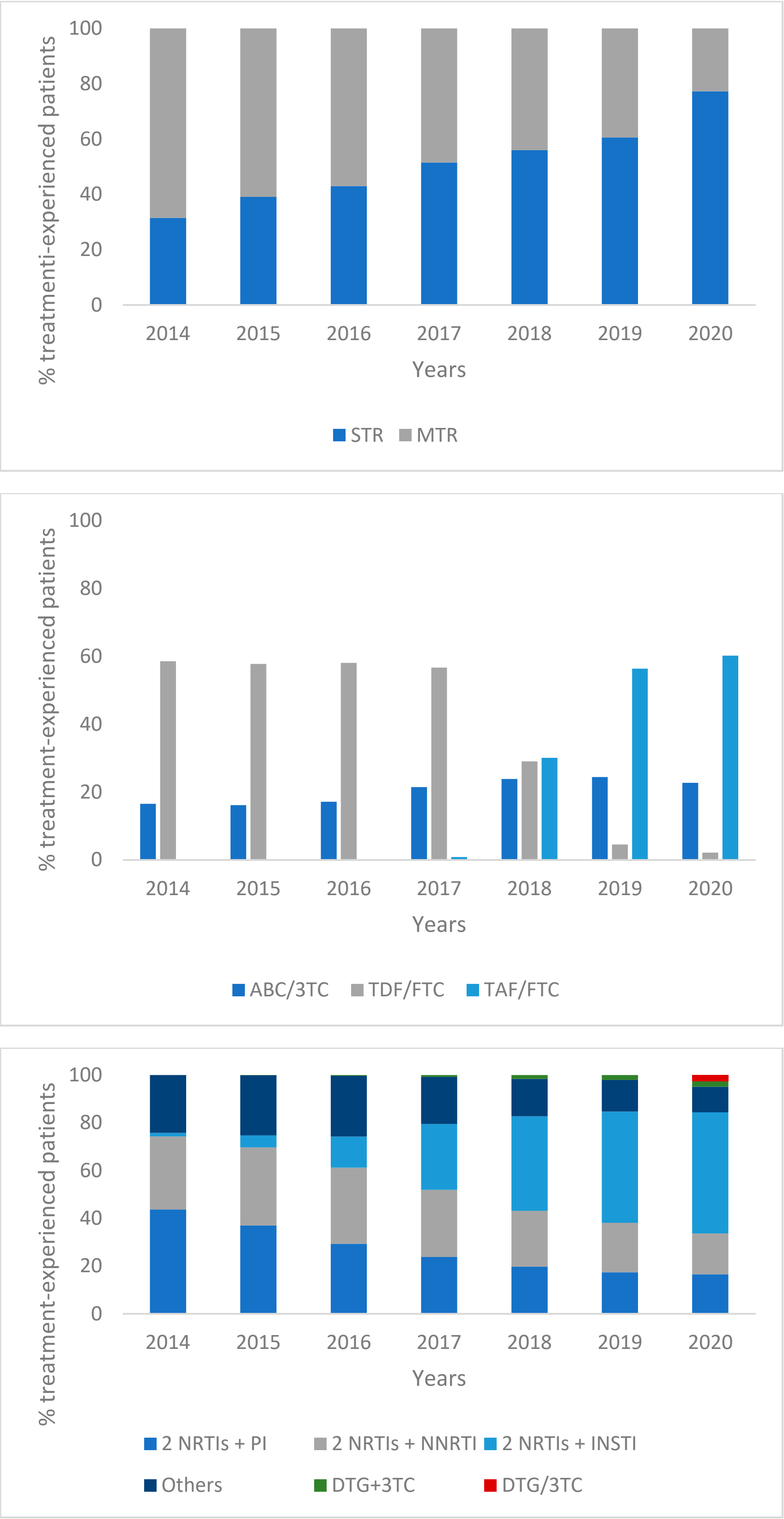

(a)

(b)

(c)

Figure S2. Distribution of treatment-experienced patients by year (2014-2020) and STR/MTR (a), NRTI backbones (b), ART regimens (c).

Abbrevation: 3TC, lamivudine; ABC, abacavir; DTG, dolutegravir; ART, combination antiretroviral treatment; FTC, emtricitabine; INSTI, integrase strand transfer inhibitor; MTR, Multiple Tablet Regimen; NRTI, nucleos(t)ide reverse transcriptase inhibitors; NNRTI, non-nucleoside reverse transcriptase inhibitors; PI, protease inhibitor; STR, Single Tablet Regimen; TAF, tenofovir alafenamide; TDF, tenofovir disoproxil fumarate.
